# The Evidence-Based Cancer Control Programs website – a strategy for disseminating effective interventions

**DOI:** 10.1186/s12875-026-03591-0

**Published:** 2026-10-23

**Authors:** Aubrey Villalobos, Jennifer Damonte, David A. Chambers

**Affiliations:** National Cancer Institute, 9609 Medical Center Drive, Rockville, MD 20850 USA

**Keywords:** Dissemination and implementation, Evidence-based interventions, Cancer prevention and control

## Abstract

The National Cancer Institute supports the Evidence-Based Cancer Control Programs website as a strategy for disseminating effective interventions to professionals who can use them in practice, including 79 tested in clinical settings such as primary care.

## Cancer prevention and control interventions and primary care

In the past six decades, scientists focused on reducing the burden of cancer have identified numerous modifiable risk and protective factors and developed a variety of effective interventions for cancer prevention and control. These include screenings for early detection or prevention of multiple cancers—breast, cervical, colorectal, lung, prostate—as well as interventions for risk reduction related to tobacco and alcohol use, diet and nutrition, physical activity, obesity management, HPV vaccination, and UV light exposure. However, it takes new evidence-based interventions (EBI) about 15 years to reach 50% uptake in practice, limiting their reach and impact [1]. Approximately 40% of cancer cases are attributable to modifiable risk factors [2]. Improving modifiable risk factors and cancer screening rates are the principal opportunities required to reduce cancer incidence and death rates [3]. Even small increases in cancer screening rates could significantly reduce population-level mortality [4]. As a highly trusted and accessible source of healthcare services, primary care providers have a critical role and great responsibility to promote these cancer prevention and early detection measures through patient education, shared decision making, and referral to resources or follow-up care.

Primary care is a uniquely well-positioned setting for implementing cancer prevention and control EBIs as most U.S. adults have an established primary care physician as their usual source of care [5]. A clinician’s recommendation is one of the most consistently identified drivers of patient adherence to cancer screening, with providers’ communication quality shown to influence uptake more than many other factors [6]. Primary care’s model of continuity enables prevention and screening messaging to be revisited and reinforced across repeated visits over time. Further, existing care team infrastructure in primary care settings, including nurses, medical assistants, and care coordinators, offers built-in capacity to help deliver EBI components.

## National Cancer Institute dissemination of EBIs

To accelerate the translation of EBIs to clinical and community practice, the National Cancer Institute (NCI) has invested for more than twenty years in a dissemination platform. Originally called *Research-Tested Intervention Programs (RTIPs)*, the online platform became *Evidence-Based Cancer Control Programs (EBCCP) *in 2020. NCI launched RTIPs in 2003 to provide broad access to cancer control intervention program materials from research studies. Details of the RTIPs evaluation and user-centered re-design are reported elsewhere [7]. At its core, EBCCP remains a searchable database of evidence-based cancer control intervention programs and is designed to provide practitioners easy and immediate access to ready-to-implement intervention materials. The program areas included on EBCCP are driven by published U.S. Federal guidelines and recommendations such as those from the U.S. Preventive Services Task Force, Community Preventive Services Task Force (The Community Guide), Advisory Committee on Immunization Practices, Surgeon General’s Advisories, Dietary and Physical Activity Guidelines for Americans, and in the absence of a Federal statement, systematic reviews and meta-analyses.

Intervention programs included on EBCCP go through a rigorous registration and review process to ensure they meet minimum eligibility criteria.^1^ These include having published peer-reviewed, statistically significant primary outcomes from an experimental or quasi-experimental study conducted within the last ten years, with available intervention materials that can be used in a U.S. community or clinical setting. Interventions on EBCCP are largely focused on enabling behavior changes; eligible outcomes are primarily behavioral and occasionally psychosocial. Intervention developers may submit their EBI for consideration and NCI conducts rolling systematic literature scans to identify newly published EBIs.^2^ After engaging intervention developers to confirm intervention eligibility and dissemination interest, NCI facilitates an external peer review of the intervention study’s research integrity, intervention impact, dissemination capability, as well as scoring its reach, effectiveness, adoption, and implementation and the readability of written intervention materials. NCI works with the study team to produce an intervention summary with implementation guidance before posting on EBCCP.

As of September 2026, EBCCP houses 177 active and 61 archived EBIs across fifteen topic areas. The number of intervention programs varies depending on several factors including when the topic was first added on RTIPs or EBCCP; for example, tobacco control, with the most interventions, was the first program area when RTIPs launched in 2003 while alcohol use and lung cancer screening were added to EBCCP in 2025 and only have one intervention each so far. EBCCP users can select filters to identify relevant EBIs including by population focus, suitable setting, socioecological level of change, EBI focus (e.g., motivation for behavior change, patient navigation, policy change), community type, and intervention target (age, sex, and race/ethnicity). EBCCP Interventions are all available in English, with many including translated materials in other languages—26 in Spanish, 5 in Vietnamese, 1 in Arabic, 1 in Swahili, and 13 other languages. Table 1 shows the number of EBIs in each program area, as well as a subset of 79 EBIs evaluated within health care facilities spanning routine cancer screening (colorectal, cervical, breast, lung, prostate), HPV vaccination, and general lifestyle/obesity counseling programs. Twenty eight of the 79 EBIs (35%) were designed for primary care delivery (i.e., embedded within family medicine, general internal medicine, or pediatric practice workflows, routine preventive/well visits, and EHR panels); 8 EBIs (10%) apply hybrid models, partnering clinic encounters with community health workers or opportunistic non-visit delivery like during Flu shot clinics; and 43 EBIs (54%) operate within specialty oncology, cancer survivorship, specialized dental, or tertiary hospital settings.

**Table 1:** EBCCP program areas and number of interventions in each category

Program Area	Number of Interventions*	Interventions Tested in Clinical Settings^†^	Primary Care Workflow Interventions^a^	Primary Care/ Community Hybrid Interventions^b^	Primary Care Clinical Scope & Example Interventions
Alcohol use	1	0	0	0	N/A
Breast cancer screening	23	13	6	2	In-clinic mammography decision aids, reminder workflows
Cervical cancer screening	14	6	1	1	Routine clinic Pap/HPV tracking, health center navigation
Colorectal cancer screening	22	18	8	3	Mailed FIT registries, EHR clinical prompts, shared decision aids
Diet and nutrition	28	2	0	1	Multi-setting lifestyle counseling
Follow-up to abnormal screening results	6	6	1	1	Primary care abnormal tracking registry
Genetic and genomic testing	3	2	0	1	Family history risk assessment tool
HPV vaccination	6	5	3	0	Adolescent well-child workflows, provider recommendation training
Lung cancer screening	1	1	1	0	Primary care LDCT shared decision-making tools
Obesity management	14	3	1	1	Pediatric well-child screening and counseling
Physical activity	32	4	1	0	Provider-delivered physical activity counseling
Prostate cancer screening	4	2	1	0	Primary care shared decision-making aid
Sun safety and indoor tanning	18	8	2	2	Well-child sun-protection counseling
Survivorship	20	13	0	0	N/A
Tobacco control	41	11	3	0	Pediatric smoke exposure, adolescent preventive visits, maternal cessation

*Program areas are not mutually exclusive (i.e., some interventions are coded to multiple program areas – e.g., combined diet and physical activity, or combined breast and cervical screening; N = 177 total unique EBIs on EBCCP as of September 2026).

^†^Interventions Tested in Clinical Settings (i.e., health care facilities, n = 79 unique EBIs). Clinical interventions tested outside of primary care or primary care/community hybrid settings include specialized oncology clinics, post-treatment survivorship programs, psycho-oncology practices, specialty diagnostic imaging/endoscopy centers, dental surgical suites, or inpatient hospital units (n = 43 unique EBIs).

^a^Primary Care Workflow Interventions (n = 28 unique EBIs): Interventions designed specifically for, and tested within, routine outpatient primary care settings (family medicine, general internal medicine, ambulatory pediatrics, Community Health Centers/FQHCs). Operational components integrated into regular clinician encounters, routine health maintenance/well-child visits, care team workflows (e.g., medical assistant/nurse prompts), or primary care panel-management EHR registries.

^b^Primary Care/ Community Hybrid Interventions (n = 8 unique EBIs): Multi-component or multi-setting interventions that link primary care clinics to community-based outreach, community health workers (promotoras), schools, or opportunistic non-visit clinical settings (e.g., drop-in influenza vaccination clinics such as Flu-FIT)

## Opportunities for dissemination and implementation of cancer prevention and control EBIs in primary care

Although our ability to collect detailed user information as a government website is limited, we have some evidence that the 2020 user-centered redesign and rebrand has increased dissemination of EBIs. Approximately 12,000 users visit EBCCP per month, based on internal webpage Google Analytics, up from fewer than 4,000 monthly users on RTIPs in 2019, with further improvements in webpage bounce rates and increases in downloads of intervention program materials. A new feature in the redesign, we now have a growing library of “Insights from the Field” case studies^3^ reporting on real-world implementation of EBCCP interventions to add practice-based evidence and data on EBI adaptations to help other users make decisions about intervention adoption. Further dissemination of EBCCP through partnerships and professional networks could expand its reach even more. However, broadening EBCCP’s reach in primary care requires more than dissemination alone; competing clinical priorities, limited visit time, the burden of integrating new workflows into EHRs, and reimbursement constraints can all limit EBI uptake even where relevant interventions are readily available on EBCCP. A recent systematic review and meta-analysis found that implementation strategies such as team-based care changes, electronic registry and reminder systems, practice facilitation, and multicomponent approaches can meaningfully increase delivery of preventive care in primary care settings [8], offering concrete avenues through which EBCCP-listed interventions might be more effectively integrated into practice.

Some have begun to leverage EBCCP to inform research directions and several cancer centers have partnered with community groups on the implementation of EBCCP EBIs locally [9–12]. Additionally, although evidence-based primary care counseling interventions for alcohol reduction, dietary habits, and adult weight management are well-documented in the broader clinical literature, their relative scarcity on EBCCP presents a scientific opportunity for implementation researchers to study their workflow integration and sustainment in busy primary care practices. Investigators may also find opportunities with EBCCP interventions to study other implementation processes (e.g., adaptation, maintenance, scale up) and possible future de-implementation of specific interventions when evidence indicates another intervention may be more effective, or evidence no longer supports its use in specific populations [13].

It is important to keep EBCCP content dynamic to encourage return users. In addition to an ongoing process for archiving outdated EBIs [7], we work with intervention developers on a rolling basis to update available EBIs, collecting new publications and information about adaptations. We also aim to publish new intervention program summaries on a regular basis but have been limited by budgetary constraints, individual EBI eligibility, and intervention developer willingness to disseminate their intervention materials. Opportunities remain to develop or adapt and rigorously test interventions in primary care settings to bolster the number of cancer screening and prevention EBIs disseminated. Development or adaptation of EBIs for primary care settings should be led by or undertaken collaboratively with representatives from the intended delivery settings following principles of authentic community engagement. To be eligible, studies need a comparison or control group and sufficient sample size to detect statistical and meaningful positive outcomes. Interventions for EBCCP should be designed with dissemination in mind [14], therefore fairly easy to replicate or adapt while retaining effectiveness.^4^ In particular, we would welcome intervention developers to submit EBIs for primary care settings in the program areas with fewest EBIs: alcohol use, lung cancer screening, nutrition and physical activity, obesity management, and genetic testing, among others.

## Conclusion

EBCCP has shown promise as a strategy for disseminating EBIs. To disseminate as many eligible EBIs as possible and ensure continued relevance of active interventions, NCI invites intervention developers to submit their outcomes papers and seeks information about EBCCP EBIs that are being used in practice for case studies. We welcome feedback and collaboration from investigators and implementation partners alike on usability of the website and use of EBIs in practice towards our goal of continuous improvement in serving the public’s health.

## Data Availability

Not applicable.

## Footnotes

1 See https://ebccp.cancercontrol.cancer.gov/eligibilityCriteria.do for details.

2 Learn more here: https://ebccp.cancercontrol.cancer.gov/programSubmissionProcess.do.

3 See https://ebccp.cancercontrol.cancer.gov/insights.do for details.

4 Learn more here: https://ebccp.cancercontrol.cancer.gov/assets/ebccp/reference/adaptation_guidelines.pdf.

## Abbreviations

EBCCP: Evidence Based Cancer Control Programs
EBIs: evidence-based interventions
EHR: electronic health record
FIT: fecal immunochemical test
HPV: human papillomavirus
NCI: National Cancer Institute
RTIPs: Research-Tested Intervention Programs
UV: ultraviolet
